# Surmounting Cancer Drug Resistance: New Perspective on RNA-Binding Proteins

**DOI:** 10.3390/ph16081114

**Published:** 2023-08-07

**Authors:** Yiyuan Feng, Sha Zhu, Tengwen Liu, Guoguo Zhi, Bingjie Shao, Jibin Liu, Baixue Li, Cen Jiang, Quansheng Feng, Peijie Wu, Dong Wang

**Affiliations:** School of Basic Medical Sciences and State Key Laboratory of Southwestern Chinese Medicine Resources, Chengdu University of Traditional Chinese Medicine, Chengdu 611137, China; yoravina@163.com (Y.F.); zhusha055@163.com (S.Z.); liutengwen_1993@163.com (T.L.); zhiguoguo2022@163.com (G.Z.); shaobingjie2023@163.com (B.S.); eaeas12@163.com (J.L.); baixuelee@163.com (B.L.); jiangcen517@163.com (C.J.); fengqs118@163.com (Q.F.)

**Keywords:** RNA-binding proteins, cancer, drug resistance, therapy

## Abstract

RNA-binding proteins (RBPs), being pivotal elements in both physiological and pathological processes, possess the ability to directly impact RNA, thereby exerting a profound influence on cellular life. Furthermore, the dysregulation of RBPs not only induces alterations in the expression levels of genes associated with cancer but also impairs the occurrence of post-transcriptional regulatory mechanisms. Consequently, these circumstances can give rise to aberrations in cellular processes, ultimately resulting in alterations within the proteome. An aberrant proteome can disrupt the equilibrium between oncogenes and tumor suppressor genes, promoting cancer progression. Given their significant role in modulating gene expression and post-transcriptional regulation, directing therapeutic interventions towards RBPs represents a viable strategy for combating drug resistance in cancer treatment. RBPs possess significant potential as diagnostic and prognostic markers for diverse cancer types. Gaining comprehensive insights into the structure and functionality of RBPs, along with delving deeper into the molecular mechanisms underlying RBPs in tumor drug resistance, can enhance cancer treatment strategies and augment the prognostic outcomes for individuals afflicted with cancer.

## 1. Introduction

**Figure 1:**
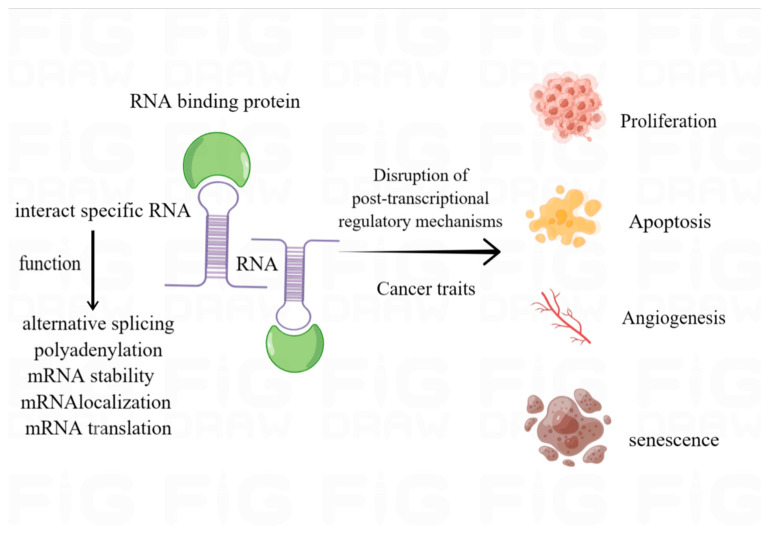
By binding to specific RNA structural domains, RBP interacts with RNA to fulfill its specific biological functions. RBP further disrupts post-transcriptional regulatory mechanisms, thereby impacting cancer progression.

Alterations in gene regulatory networks cause changes in the cellular proteome, leading to altered phenotypes and abnormal cell proliferation. Abnormal gene expression and abnormal regulatory functions in cells are responsible for the development of cancer. Typically, this is thought to be associated with mutations and translocations of cancer-related genes. However, recent studies have shown that a group of cancer-associated RBPs can regulate the expression of specific genes in cells by interfering with post-transcriptional mechanisms such as mRNA stability and translation, thereby contributing to the development and progression of cancer [1,2,3] (Figure 1).

The mediators that influence the occurrence of post-transcriptional regulatory mechanisms are cis-acting elements, mainly found in the 5′- and 3′-untranslated regions (UTR) of mRNA, and trans-acting transcription factors that can specifically recognize and interact with cis-acting elements [4,5]. Among them, trans-acting transcription factors mainly include RBPs and non-coding RNAs (ncRNAs), such as microRNAs (miRNAs) and long-stranded non-coding RNAs (LncRNAs) [6]. Different types of RBP regulatory networks can regulate post-transcriptional mechanisms such as mRNA stability and translation by interfering with regular sequence motifs and RNA secondary structure, thereby regulating the expression levels of cancer-related genes [7]. Various RBPs have been found to regulate a wide range of genes associated with cancer development.

RBPs are highly conserved and abundant proteins that play a key role in maintaining normal and stable gene expression and are key players in gene expression and a variety of post-transcriptional regulatory mechanisms involved in various aspects of RNA regulation [8,9], including transcription, translation [10], splicing [11], polyadenylation [12], stability [13], and localization [14]. RBPs can form ribonucleoprotein complexes (RNPs) through direct interactions with other proteins or as scaffolds for coding or ncRNAs, and the life cycle of RNAs can be affected by the interaction of RBPs with other proteins, resulting in abnormal protein phenotypes that contribute to tumorigenesis and progression [15].

There are approximately 1914 RBP genes in the human genome, accounting for 7.5% of protein-coding genes [15,16]. When cancer occurs, many RBP confinements are opened, and the expression levels and localization of RBPs are altered, resulting in changes in the expression levels of proto-oncogenes, oncogenes, and genomic stability-related genes. For example, the expression levels of cancer-related genes such as CD44 and VEGF are affected by changes in RBPs as splicing factors. Also, based on genome-wide analysis, as they largely affect cell growth and proliferation, many RBPs are considered to be key factors in cancer development and progression. The majority of RBPs are comprised of one or more RNA-binding domains (RBDs), such as RRMs, KH structural domains, dsRBDs, znFs domains, and PAZ structural domains [17]. For instance, hnRNPA1 possesses RRMs and hnRNP, IGF2BPS contains RRMs and KHs, and ZEB1 is composed of ZnF-CCCHs and ZnF-CCHCs. The diverse arrangements of these RBD combinations enable RBPs to regulate various mRNA functions [18].

Overall, the broad function of RBPs confers a central role in the RBP-RNA regulatory network during disease progression, particularly in cancer [19]. Thus, exploring the mechanisms of drug resistance induced by RBP abnormalities may provide potential approaches to improving cancer prognosis. This paper reviews the function and structure of RBPs, highlights the regulatory role of RBPs in therapeutic resistance, and discusses RBPs as potential targets for cancer therapy.

## 2. Definition of RBPs and Their Structural Features

RBPs are proteins that bind directly to the RBD in RNA to form RNPs, which are directly involved in cellular life activities such as synthesis, processing, translocation, translation, degradation, and function of coding and non-coding RNAs [20]. RBP ensures that genetic information can be expressed as protein through DNA and RNA, which means that RBP is essential for all physiological and pathological processes [21].

**Figure 2:**
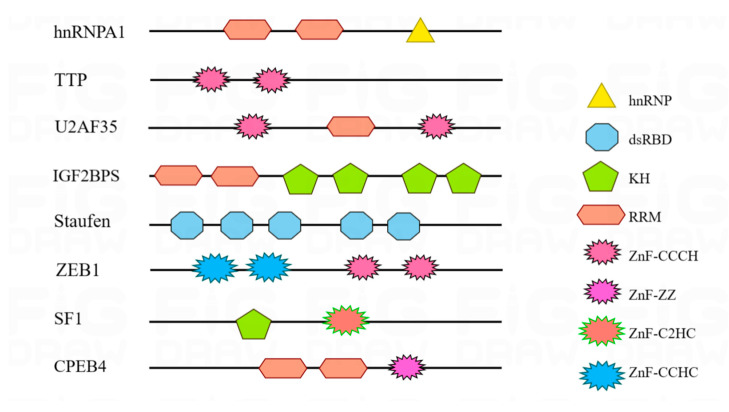
Several common RBDs are represented by different shapes and colors, as shown in the diagram. Examples include RRM, KH structural domains, DsRBD, and ZnFs domains. One or more RBDs are arranged and combined in different ways to form RBPs with different functions, which ultimately bind specifically to the target RNA.

The RBD is the functional unit that forms the RBP. The presence of one or more typical RBDs in a specific order ensures that RBPs can bind properly to RNA. RBPs can be classified as canonical or non-canonical according to the presence or absence of an RBD, and we refer to proteins containing RBDs as canonical RBPs. It has been shown that many RBPs lack a classical RBD. The absence of a classical RBD leads to structural instability of the RBP, which affects the ability of the RBP to bind to RNA and, thus, the stability and translation of mRNA [22]. Many RBDs are very small (<100 residues) and only use a small number of residues to interact directly with RNA. A typical and well-structured RBP consists of multiple repetitive sequences that contain only a few specific basic domains. These repetitive sequences can be arranged in different combinations with specific RNAs, thus allowing for a diversity of RBP functions [18]. Furthermore, precise recognition of proteins can be achieved by rearranging these typical structural domains [23]. Here we present several classical RBDs, including RRM, KH structural domains, dsRBDs, and znFs domains (Figure 2).

### 2.1. RNA Recognition Motifs (RRM)

RRM, also known as the RBD or ribonucleoprotein motif RNP, is by far the most common and well-studied RNA binding module. A typical RRM consists of 80–90 amino acids and has a βαββββαβ topology, forming two alpha helices against a four-stranded anti-parallel beta-sheet. Over 10,000 RRMs have been identified, and approximately 0.5–1% of all human genes contain RRMs. Most act during post-transcriptional gene expression, where RNA recognition occurs [23]. In most cases, recognition usually occurs on the surface of the β-sheet, mediating post-recognition binding through three conserved residues: an Arg/Lys salt bridge forming the phosphodiester backbone and two aromatic residues that create stacking interactions with the nucleobase. RRM-containing proteins bind to different RNAs by recognizing two to eight nucleotides of single-stranded RNA; a single RRM can recognize four to eight nucleotides by using exposed loops and other secondary structure elements not present in the canonical structure [24,25,26], and this general recognition mechanism is present in many but not all RRMs [27,28], with some of these structural domains even interacting with proteins rather than RNA [29,30,31,32,33,34]. Some individual RRMs can bind to RNA with high specificity, but in many cases, multiple structural domains are required to define specificity because the number of nucleotides recognized by a single RRM is usually too small to define a unique binding sequence.

### 2.2. The K Homology (KH) Domain

The K-Homology domain is a single-strand, sequence-specific nucleic acid binding domain first identified in heterogeneous ribonucleoprotein K (hnRNPK) and commonly found in proteins that regulate gene expression in eukaryotes and prokaryotes [35]. Compared with RRM, the KH structural domain is smaller, consisting of 70 amino acids, and is located at the center of a very functionally important structural domain. Typically, this structural domain has a conserved GXXG loop, which is able to link the two α-helices and the β-strand [18], recognizing four nucleotides in ssRNA or ssDNA. All KH structural domains form a three-stranded β-sheet stacked on three α-helices. Based on their topology, they can be divided into two subfamilies (type I: βααβββα topology; type II: αβββααβ topology [36]). As with RRMs, individual RBPs with repeating KH structural domains can increase the chance of binding. These KH structural domains are also able to increase binding specificity independently or in concert [37].

### 2.3. Double-Stranded RNA-Binding Structural Domains (DsRBDs)

DsRBDs or motifs (dsRBMs) are the third most common RBDs, consisting of 65–70 amino acids [38], with amino acids folding into an α1β1β2β3α2 structure, forming an antiparallel β-sheet and laterally attached to an α-helix on one face. DsRBDs play a role in viral protection, RNAi, and cellular transport of proteins found in proteins such as ribonuclease III and RNA editing enzymes. DsRBD has a central role in binding double-stranded RNA (dsRNA) or highly structured RNA, but it does not make specific contact with nucleobases [38]. DsRBD usually occurs as a tandem repeat sequence or in combination with other functional RNA-binding structural domains. Unlike the RRM and KH structural domains, most intermolecular contacts of the dsRBD are sequence independent, forming hydrogen bond contacts with the 2′-OH group and the phosphate backbone 46 [39]. The presence of multiple dsRBDs can confer specificity to specific structures as they are able to recognize certain arrangements of the RNA helix [40,41,42]. Stacking interactions are rarely seen in dsRBDs, a phenomenon that provides some evidence to explain the low affinity of dsRBDs for RNA targets [43,44].

### 2.4. Zinc Finger Structural Domains (ZnFs)

ZnFs are classical DNA-binding proteins that can also bind to RNA, proteins, and small molecules [45]. ZnFs comprise a large family of proteins, with members averaging 30 amino acids in size. ZnFs have the ability to form a simple ββα topology, and Zn^2+^ is able to coordinate residues in the β hairpin of this structure with the α-helix for alignment [46]. ZnF isoforms that interact with RNA include the CCHC (zinc finger joints), CCCH, CCCC (RanBP2), and CCHH isoforms, where the cysteine (C) and histidine (H) arrangement determines the RNA binding preferences of the ZnFs, displaying a range of sequence and structural specificities [25,47]. ZnFs can use some of the same residues to recognize both nucleic acids, but different DNA and RNA structures dictate different structural arrangements of ZnFs on nucleic acid templates. Zinc finger joints (CCHC) recognize stem-loop elements in RNA (or ssDNA) by contacting bases in the loop and the phosphate backbone of the stem. Subtypes with multiple CCCH and CCCC tend to bind the 3 nucleotide repeats of RNA, whereas subtypes with abundant CCHH interact with single- and double-stranded RNA and dsRNA. CCHH ZnFs interact with DNA primarily by forming direct hydrogen bonds with Watson-Crick base pairs, using them to recognize residues in α-helix 60 [48]. In contrast, TFIIIA binds RNA by specific contact with two RNA loops through the recognition helices of fingers 4 and 6.

## 3. The Function of RBP and Its Aberrant Expression in Cancer

### 3.1. Function of RBP

#### 3.1.1. Pre-mRNA Alternative Splicing (AS)

**Figure 3:**
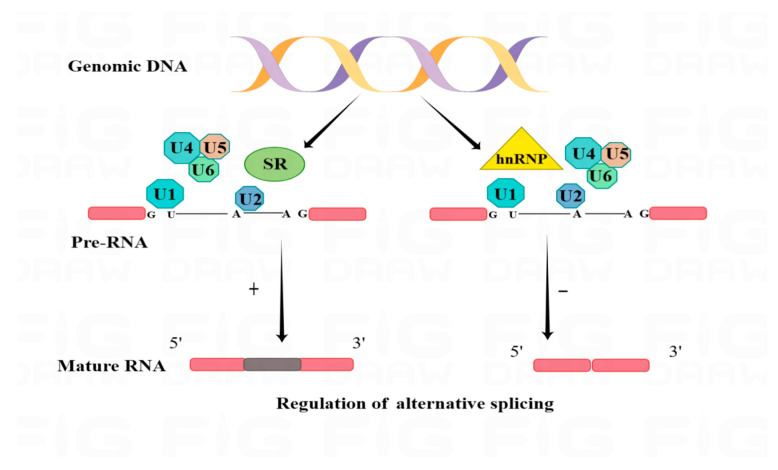
RBPs form complexes with snRNP and polypeptides that together regulate the process of selective splicing. U1-U6 represent spliceosomal components, or snRNPs, that bind to the 5′ and 3′ splice sites. As regulators, the RBP, SR protein family, and hnRNP proteins exert an enhancing (+) or inhibiting (−) effect on exon inclusion, respectively, as indicated by the arrows.

The core spliceosome is a key regulator of selective splicing and is composed of small nuclear proteins (snRNPs), peptides, and RBPs. RBPs can form trans-acting splicing factors with cis-regulatory precursors to regulate splicing events [49]. The most representative splicing regulators are the serine/arginine-rich (SR) family of proteins and the hnRNP proteins, which affect the recruitment of spliceosomal components and the selection of splice sites by recognizing and binding exons for enhancer splicing and by participating in RNA-protein interactions [50]. HnRNP proteins act as another splicing regulator, mainly by inhibiting the splicing process by disrupting exon recognition [51] (Figure 3).

#### 3.1.2. Alternative Polyadenylation of mRNA (APA)

**Figure 4:**
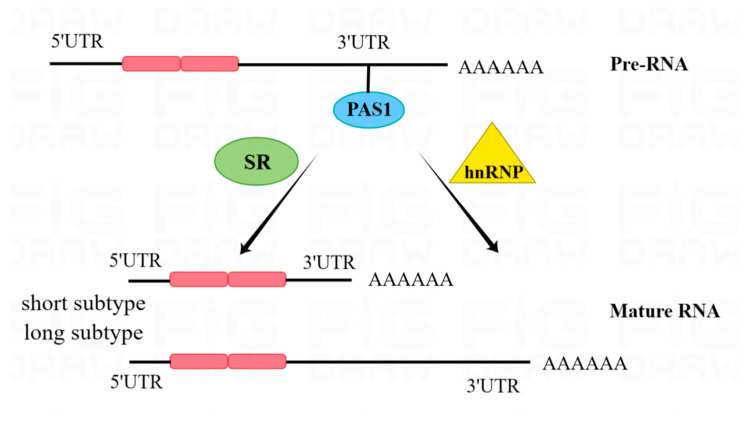
The important site where alternative polyadenylation occurs is at the mRNA 3′-UTR, where RBPs enhance (+) or inhibit (−) the use of PAS sites, resulting in 3′-UTRs of different lengths.

APA is a key component in the generation of mature RNA transcripts, and the mRNA 3′-UTR is an important site where APA occurs. In addition, APA can mediate 3′-end cleavage and polyadenylation (CPA), resulting in 3′-UTRs of variable length [17]. RBP is a major component of the APA machinery, which includes cleavage stimulating factors, cleavage factor I, and cleavage factor II, among others. In addition to direct involvement in the APA pathway, RBPs can recruit or compete with polyadenylation-associated proteins to regulate the CPA process in target mRNAs [52]. HnRNP and SR family proteins can also regulate the polyadenylation process in mRNAs [53] (Figure 4).

#### 3.1.3. RNA Stability

**Figure 5:**
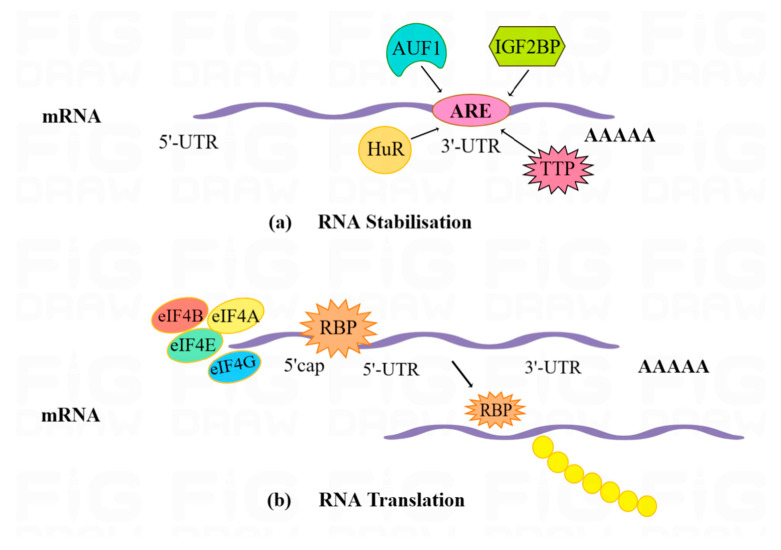
(**a**). RBPs such as AUF1, HuR, TTP, and IGF2BP family proteins regulate the stability of target RNAs by binding to the ARE sequence element of the 3′-UTR in mRNA. (**b**). The mRNA translation process is dominated by the 5′cap structure of the eIF4 cap-binding complex, which recognizes the relevant elements in the target RNA structure and thus efficiently synthesizes the protein.

The 5′ terminal 7-methylguanosine (m^7^G) cap and the 3′ poly (A) tail of RNA determine the stability of RNA, which is regulated by RBPs [54,55]. ARE-associated RBPs such as AUF1 (AU-rich elemental RNA-binding protein 1), HuR (Human antigen R), TTP (trite-traproline), IGF2BP (Insulin-like growth factor 2 mRNA-binding protein) family proteins, and Wig1 can recognize and bind to AREs, thereby regulating the stability of target mRNAs [56] (Figure 5a).

#### 3.1.4. RNA Localization

**Figure 6:**
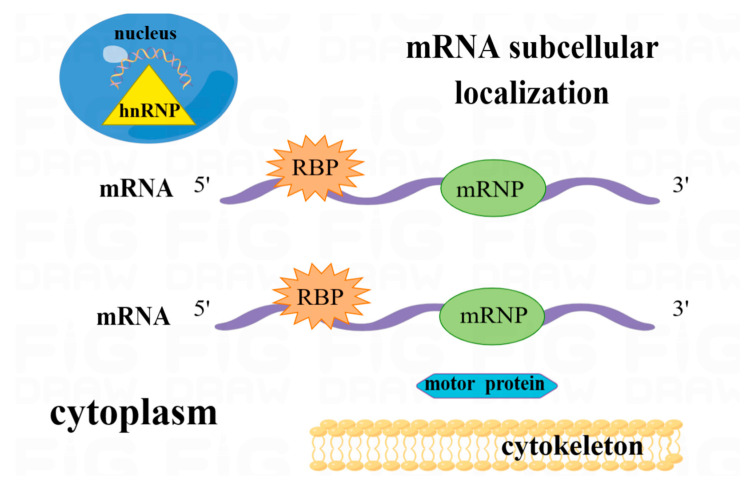
HnRNP assists in the translocation of pre-mRNA into the cytoplasm, which then enters the cytoplasm and becomes mRNA, a substrate for mRNA localization. RBP binds to pre-mRNA to form mRNA. RBPs assemble into multiunit complexes that bind RNA to the cytoskeleton and protein motors, ensuring that mRNA can be efficiently transported to the target site.

RBP binding to RNA coordinates the localization of target RNA and its translation location, which is essential for the regulation of RNA stability and translation. RBPs recognize and bind to specific sequences in the target RNA, and the bound RBPs subsequently assemble into multiunit complexes that bind the RNA to cytoskeletal molecular motors and transport it to the target site (Figure 6).

#### 3.1.5. Translation

RBPs are dynamic components of ribonucleoproteins, which are key factors in the process of cellular translation. Different translation-associated RBPs bind differently to mRNAs, resulting in different translation efficiencies [57]. RBPs promote translation by recognizing relevant elements in the structure of the target RNA [58,59,60,61] (Figure 5b).

### 3.2. Aberrant Expression in Cancer

Over the past few decades, studies have shown that dysregulation of RBPs contributes to cancer treatment resistance. RBPs can target many target RNAs to influence the characteristics of cancer cells. RBPs can bind not only to mRNA exons, introns, and untranslated regions (UTRs), but also to ncRNAs such as miRNAs, siRNAs (small interfering RNAs), telomerase RNAs, tRNA, small nucleolar RNA (snoRNA), and spliced small nucleolar RNA (snRNA). Upon binding to secondary structures formed by ncRNAs, RBPs are involved in regulating several processes of gene expression, such as RNA modification, protein localization and secretion, and chromosome remodeling [21]. Dysregulation of RBPs affects the expression levels of target RNAs associated with the cancer phenotype, such as proliferation, apoptosis, angiogenesis, senescence, and EMT/invasion/metastasis [17]. Dysfunctional RBPs lead to abnormalities in cellular processes, and these abnormalities cause alterations in the proteome. An abnormal proteome can cause an imbalance in the checks and balances between oncogenes, leading to cancer. For example, in human CRC (colorectal cancer) tissues, the expression of the long non-coding RNA (lncRNA) NEAT1 is upregulated, which is associated with poor prognosis in CRC patients. NEAT1 interacts with the RBP DDX5 and activates the Wnt/β-linked protein signaling pathway, which further promotes tumorigenesis [62]; the molecular chaperones required for DICER1 to function are the RBP TRBP, and mutations in TRBP can also lead to aberrant miRNA expression as well as proliferation and differentiation of cancer cells [63,64]; in addition, the epithelial-mesenchymal transition (EMT)-specific transcription factor ZEB1 protein directly inhibits the mRNA levels of epithelial splicing regulatory protein 1 (ESRP1), leading to cell surface antigen Increased expression of various spliceosomes of CD44 and induction of stem cell-like and aggressive cells in lung, breast, and pancreatic cancers. A single RBP can bind multiple target mRNAs, and different RBPs can also regulate cellular processes by sharing the same specificity on mRNAs bound to the same mRNA [65]. This complex, overlapping post-transcriptional RNA regulation works together with other cellular process-participating factors to form a more complex regulatory network that plays an irreplaceable role in the regulation of cancer-related gene expression [66].

## 4. Molecular Mechanisms of RBPs in Tumor Drug Resistance

### 4.1. Selective Splicing

The processing of pre-mRNAs interacting with RBPs, particularly selective splicing, is affected when the function of RBPs is dysfunctional [67]. Selective splicing is an important mechanism that drives transcriptome diversity, and AS alters gene expression by generating multiple mRNA transcripts from a single precursor gene, through which a variety of unique transcripts are generated and subsequently translated during cellular processes [68,69,70]. Dysregulation of AS is extremely common in the development and progression of cancer. Most of the proteins involved in the AS process are RBPs, which bind to target RNAs and thus influence the splicing process [71,72,73]. Different species of RBPs, such as SRSF, hnRNPs, PTB, ESRP, and QKI, can exert positive or negative regulatory effects on selective splicing. Through selective splicing processing, pre-mRNAs can be transcribed into a variety of mRNA variants with different stability and protein-coding potential. Different mRNA splice variants contribute to increased protein diversity in cancer [74]. In cancer cells, aberrant RBP expression and aberrant RNA splicing are observed more frequently compared with normal cells [75], and these alterations lead to changes in the expression levels of proto-oncogenes and oncogenes, thus promoting cancer development and progression [49,74,76].

In a variety of cancers (including HCC), RBP abnormalities usually lead to a number of abnormal AS events. Examples include increased or decreased exons and dysregulation of splicing factors [68,77,78]. Also, dysfunction of hnRNP and SR can be observed in a variety of cancers, suggesting a key role for selective splicing in cancer progression. The complexes that determine tissue- and tumor-specific splicing events are formed by the association of core proteins of the splicing machinery with RBPs, and these complexes can collaborate with or antagonize spliceosomal activity depending on the position of the RBPs binding site in relation to the regulated exon.

### 4.2. Polyadenylation

Selective polyadenylation is an extremely critical step in the generation of mature RNA transcripts [79]. APA occurs mainly within the 3′-UTR of mRNA and produces 3′-UTRs of different lengths through 3′-end CPA [80]. APA can regulate the stability, subcellular localization, and translation efficiency of target mRNAs by altering the 3′-UTR length [81]. This tissue-specific mechanism can convert the coding sequence of transcripts to produce different isoforms, affecting their metabolism in various ways, including stability, nuclear export, and cellular localization [82]. RBPs can regulate the CPA of target mRNAs by recruiting or competing with APA machinery proteins [52], thereby affecting APA progression. Similar to selective splicing, selective polyadenylation enables a single gene to have encoded sequences or different transcripts in the untranslated region and can affect the function of the protein encoded by that gene by altering the coding sequence.

The C-terminal region of CPEB family proteins contains two RRMs and two zinc finger-like motifs, while CPEB family proteins also possess a variable N-terminal region [83]. CPEB 1–4 can regulate the length of the poly (A) tail of mRNA using cytoplasmic polyadenylation elements (CPE), resulting in a shorter 3′-UTR for transcripts associated with cell proliferation and tumorigenesis. CPEB 1 regulates nuclear transcription-specific APA and can modulate cytoplasmic polyadenylation-mediated translation [84]. If CPEB1 is absent, the poly (A) tail of mRNA is prolonged, and mRNA translation of MMP9 is enhanced in breast cancer cells [85].

### 4.3. Stability

The stability of eukaryotic RNA is ensured by two stability determinants: the m^7^G cap at the 5′ end and the 3′ poly (A) tail. The normal process of RNA degradation is an essential part of the gene expression process. The 3′-poly (A) tail and the adulterated 5′ cap protect the structure and stability of eukaryotic RNA well [86]. These two components protect the mRNA from decay and facilitate the initiation of translation [54]. In order to initiate decay, either of these structures must be disrupted, including deacetylation of the poly (A) tail, removal of the 5′ end m^7^G cap (decamp), or the mRNA, which must be cleaved internally by a nuclease-mediated reaction. The ARE is a structure that has been extensively studied as being closely related to mRNA stability [87] and is present in approximately 16% of transcripts [22,88]. Typically, cellular transcripts containing AREs are inherently unstable. The most common cis-unstable element in the 3′-UTR is the ARE, which achieves decay of mRNA mainly through deacetylase-mediated shortening of the poly (A) tail. The ARE-binding protein (AUBP) is actively involved in this destabilization process. Based on different cellular stimuli, AUBP is involved in destabilization or participates in stabilization [89].

In cancer, these ARE-containing transcripts appear to overexpress oncogenes, growth factors and their receptors, inflammatory mediators, and cell cycle genes, among others. This phenomenon suggests that mRNA stability plays an important role in cancer production and metastasis [90,91]. For mRNA stability, RBPs have a bidirectional regulatory role. For example, AUF1, HuR, PTBP, IGF2BP family proteins, and MCPIP1 can enhance mRNA stability in cancer, regulate the protein expression of their target genes, or accelerate the degradation of lncRNA in cancer [56].

The AU-rich elemental RNA-binding protein 1 (AUF1, also known as hnRNPD) contains a family of four splice isoforms that become p37AUF1, p40AUF1, p42AUF1, and p45AUF1 depending on their molecular weight [92]. AUF1 has a bidirectional role in maintaining mRNA stability in different systems. Transgenic mice overexpressing the p37AUF1 isoform develop spontaneous sarcomas, while cancer-associated transcripts such as CCND1, FOS, and MYC mRNA are significantly increased. In addition, AUF1 can trigger an anti-tumor response by destabilizing mRNAs encoding the anti-apoptotic protein BCL2 and the pro-inflammatory factors GM-CS, IL-6, IL-10, and TNF-α.

HuR expression has been reported to be extremely elevated in several cancer types [93]. Overexpression of HuR in cancer can stabilize several ARE-containing mRNAs encoding cell cycle regulators, such as CCNA 1, CCNB 1, CCND 1, and CCNE 1, which contribute to the proliferation of cancer cells. In addition to promoting HCC by stabilizing mRNA transcripts to promote cancer cell proliferation, HuR can also intervene in cancer progression by regulating RNA stability through IncRNA [94,95]. HuR can promote the growth of glioma cells through post-transcriptional regulation while enhancing their resistance to a variety of drugs, such as etoposide, topotecan, and cisplatin [96]. This process occurs mainly through HuR binding to the 2′UTR of MCL3, BCL1, and BLxL to stabilize their mRNAs, thereby regulating some genes involved in apoptosis. In contrast, mRNA levels of C-Myc, Wnt5a, and P27 were down-regulated by HuR.

The three main members of the PTBP family are PTBP1, PTBP2, and PTBP3, of which PTBP3 promotes the growth and metastasis of cancer cells and also prevents the degradation of mRNAs. This is achieved by promoting EMT in breast tumor cells and by regulating the expression of the transcription factor ZEB3 by binding to the 1′UTR of their mRNA, respectively [97].

In addition, the IGF2BP family can play an important role in the tumor development process by regulating mRNA stability. On the one hand, IGF2BP1 protects the degradation of PTEN mRNA and thus promotes the migration of tumor cells [98], while on the other hand, IGF2BP1 promotes the expression of C-Myc and MKI67 mRNA to regulate the proliferation and apoptosis of hepatocellular carcinoma cells [99]. In addition, IGF2BP1 reduces the stability of HULC mRNA, which is specifically and highly expressed in hepatocellular carcinoma. IGF2BP2 has the ability to stabilize HMGA1 mRNA and RAF196 mRNA, thereby enhancing cancer cell viability and promoting cancer cell proliferation [100]. IGF2BP3 promotes the degradation of EIF4E-BP2 mRNA, thereby promoting cervical cancer cell proliferation [101].

### 4.4. Subcellular Localization

It is well known that the subcellular localization of mRNAs or lncRNAs is closely related to their stability and translation. The biological function of lncRNAs is determined by their subcellular localization [102]. RBPs associated with cancer often bind to RNAs to coordinate their localization and translation [95,103]. RBPs typically associate transcripts with cytoskeletal molecular motors by binding to sequences in the 3′-UTR, which in turn deliver RNPs to specific subcellular compartments, thereby localizing mRNAs within the cell. This mechanism is essential for establishing and maintaining cell polarity but is frequently altered during carcinogenesis [104].

Among the CPEB family members, CPEB1 regulates the localization of ZO-1 mRNA, which encodes a key tight junction component. When CPEB1 is depleted, ZO-1 mRNA is randomly distributed, and the central lumen does not form properly, resulting in a loss of epithelial cell polarity [105].

RBP Tia1 can interact with a variety of cancer-related mRNAs and is involved in multiple aspects of cancer development and progression, such as cancer cell proliferation, apoptosis, invasion, metastasis, angiogenesis, and immune escape [106,107]. Tia1 functions to regulate the translational silencing and localization of cellular stress-related transcripts (p53 mRNA), a function that is achieved by binding to an mRNA, and this function is achieved by binding to a subset of P53 mRNA [108]. When the DNA of Tia1 is disrupted, Tia1 and mRNA dissociate, and P53 mRNA is released from the stress granule and bound to the polyplex, which causes mRNA migration and translation.

IGF2BP1 is extremely important for the development of cancer. After transcriptional processes have occurred, they influence mRNA expression in oncogenes by regulating the subcellular localization of mRNAs encoding β-actin, E-calmodulin, α-actin, and Arp-16 (components of the Arp 2/3 complex), thereby promoting tumor cell proliferation and growth, invasion, and chemical metabolism [109,110]. IGF2BP1 promotes the transfer of polar cell ACTB transcripts to actin-rich protrusions in polarized cells [98]. IGF2BP1 binds to β-actin transcripts in the nucleus and induces translational silencing of mRNAs in the cytoplasm without affecting their stability. Elevated expression of IGF2BP1 can be found in primary tumor tissues such as breast, colon, and non-small cell lung cancers [111,112,113]. In contrast, IGF2BP1 expression is reduced in metastatic cells. Reduced expression of IGF2BP1 inhibits the transport and local expression of adhesion- and migration-related target mRNAs [110,114]. When IGF2BP1 is silenced, intercellular junctions are disrupted, and cell adhesion is reduced, resulting in enhanced cell migration and invasion.

### 4.5. Translation

The translation process involves the coordination of three major components: initiation, elongation, and termination. For the regulation of mRNA translation, most interventions are made at the initiation step [115,116]. EIF4E (Eukaryotic translation initiation factor 4 E) is a 5′ cap-dependent translation initiation factor, and due to the broad function and expression pattern of translation factors and associated RBPs, the RBP of elF4E plays a crucial role in the induction of translation by facilitating the ribosome assembly and loading process [117,118,119,120]. Almost all major oncogenic signaling pathways that are altered in cancer, such as PI3K/AKT/mTOR, RAS/MAPK, and Wnt/b-linked proteins, result in translational dysregulation [57,119,120].

The translation of the oncogenic program is guided by structure- and sequence-specific regulatory elements. One of the most widely studied 5′-UTR structural elements is the internal ribosome entry site (IRES), which promotes translation in a cap-dependent manner by directly recruiting ribosomes in association with IRES trans-acting factors (ITAFs). IFAFs are composed of a set of RBPs that act on IRES RNA elements located in the 5′-UTR in a cap-independent manner, recruiting ribosomal subunits, which in turn undergo translation initiation [115,116]. The development of cancer is associated with the dysregulation of cap structure-dependent translation.

La ribonucleoprotein structural domain family member 3 (LARP 3) can promote cancer cell survival and invasion by inducing IRES-mediated translation of anti-apoptotic XIAP and EMT-associated LAMB 1 mRNAs [121,122].

EIF4E is a component of the eIF4F translation initiation complex. EIF4E interacts with eIF4A (RNA decapping enzyme) and eIF4G (scaffold molecule) and binds the 5′-terminal m^7^G cap of mRNA. EIF4F, the 5′-cap binding complex, induces mRNA cyclization and translation activation via ribosome loading. Among the components of the eIF4F complex, eIF4E is commonly overexpressed in different types of tumors, which is associated with a poor prognosis [123]. A study showed that eIF4E haploinsufficient mice were physiologically normal but significantly resistant to tumor formation [124]. This suggests that mammalian cells express eIF4E above the threshold required for normal translational control but that the factor may be upregulated by cancer cells to induce the translation of specific subgroups of oncogenic mRNAs.

HuR can enhance the translation of transcripts encoding ProTα, P53, and MSI 1 by binding to the 3β-UTR in a miRNA-independent manner, and increased cytoplasmic ProTα mRNA levels have been associated with anti-apoptotic effects in cancer cells [125,126]. Meanwhile, HuR can regulate MSI1 mRNA and its translation in glioblastoma. HuR and PTB (also known as hnRNP I) bind to the HIF-1α5 β-UTR and 3 β-UTR, respectively, thereby synergistically increasing HIF-1α translation in the presence of hypoxia [127]. In addition, HuR can negatively regulate mRNA translation in a miRNA-dependent manner [128].

Here, we summary the molecular mechanisms of RBP in the cancer (Table 1).

## 5. RBP-Targeted Cancer Therapy

### 5.1. Small Molecules

**Figure 7:**
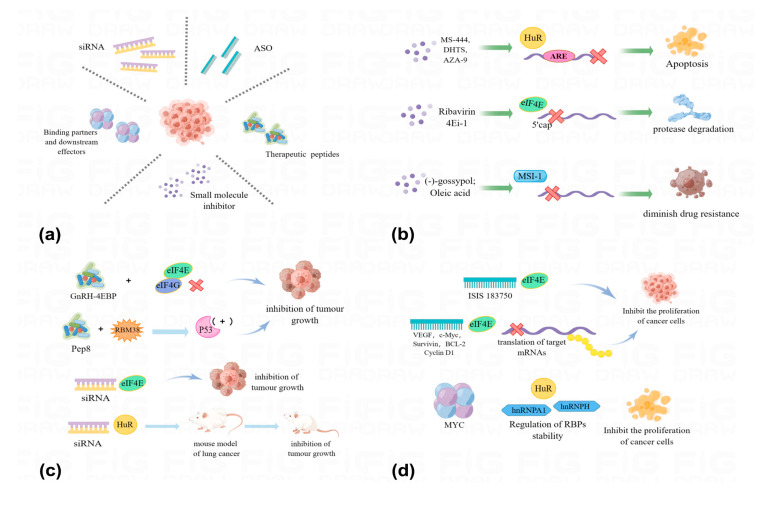
Current RBP-based targeted therapeutic strategies can be classified as either RBP-specific manipulation or RBP-RNA interactions. The cancer therapeutic approaches summarized in figure (**a**) are classified as small molecule inhibitors, therapeutic peptides, ASOs, siRNAs, RBP-binding partners, and downstream effectors. The main mechanisms are to inhibit RBP-RNA interactions by inducing degradation, inhibiting enzyme activity, blocking post-transcriptional modifications, or competing for selected RBPs through binding. Schematic representation of RBP-targeted therapeutic strategies is shown in figure. (**b**) Small molecule inhibitors, figure. (**c**) Therapeutic peptides and siRNAs, figure. (**d**) ASO, RBP-binding partners, and downstream effectors.

Cancer therapeutics targeting RBPs, represented by small-molecule inhibitors, are in high demand [159]. Small-molecule probes that target and interfere with RNA binding are new approaches that have recently been developed to block RBP function and have been characterized by researchers [160]. It has been shown that some small-molecule drugs can regulate RBP function by targeting it in various human diseases, including cancer, thus promising to improve therapeutic resistance to RBP transmission (Figure 7b).

Abnormal expression of HuR is often seen in various types of cancer, and this is closely associated with treatment resistance in cancer. A range of small-molecule inhibitors targeting HuR have been developed to alleviate treatment resistance in cancer. Among them are, for example, MS-444, DHTS, and AZA-9, nanomolecular inhibitors of HuR that block its RNA-binding activity by targeting RRM1 and RRM2 of HuR. In the presence of these inhibitors, the ARE binding activity of HuR to the target RNA is significantly reduced [161,162]. Since HuR-ARE interactions are important for the stability of many mRNAs associated with therapeutic resistance, the potential exists for the use of these inhibitors to reverse therapeutic resistance [163]. In colorectal cancer, after treatment with small molecule inhibitors, HuR is unable to bind to ARE-containing mRNA targets (e.g., IL-2, IL-1β, TNF-α, COX-2, and C-fos), its function is disrupted, and it exhibits selective anticancer effects [161,162,164,165]. In the treatment of non-small cell lung and thyroid cancer progression, CMLD-2 has been shown to be an effective HuR inhibitor that binds competitively to HuR, thereby inhibiting HuR’s cell growth and proliferation-promoting effects and the expression of related genes, increasing apoptosis in tumor cells, and thereby slowing the progression of cancer [165,166]. Wu et al. identified, by establishing a high-throughput screening system, several compounds that inhibited the activity of HuR by disrupting HuR-mRNA interactions. Therefore, these compounds could be used as cancer therapeutic agents targeting HuR [167].

EIF4E has also been associated with therapeutic resistance in a variety of cancers. Overexpression of eIF4E has been reported in a variety of cancers, which correlates with the aggressive phenotype of tumors. Ribavirin, an antiviral guanosine analog, blocks the translocation and translation of oncogenes regulated by eIF4E [168]. As another eIF4E inhibitor, N-7 benzyl guanosine monophosphate tryptamine phosphonamidite prodrug (4Ei-1) upregulates gemcitabine chemosensitivity in lung and breast cancers by inhibiting the mRNA cap-binding ability of eIF4E and degrading the eIF4E protease [169].

Musashi RNA-binding protein 1 (MSI-1) has a role in enhancing therapeutic resistance in cancer. MSI-1 enhances therapeutic resistance by increasing the expression of DNA repair-associated proteins DNA-PKcs and EGFR, and therefore cancer treatment, in a way that identifies potential inhibitors of MSI proteins using high-throughput analysis [170]. In one experiment, a natural compound called (−) -gossypol, a small molecule known to have anticancer effects on various cancers, was screened by fluorescence polarization. (−) -Gossypol can occupy the consensus RNA binding site of MSI-1, thereby disrupting the interaction between MSI-1 and its target mRNA to achieve inhibition of MSI-1 [171]. Another study found that oleic acid could interact with the RRM1 motif in MSI and induce a change in MSI conformation, thereby inhibiting MSI binding to target mRNAs. In addition, oleic acid can also influence cancer progression by upregulating MSI-1 expression levels to affect cell division [172].

### 5.2. Therapeutic Peptides

Therapeutic peptides contain 55 amino acids or less and have advantages such as high specificity, selectivity, small size, ease of modification, and biocompatibility [173]. Currently, various therapeutic peptides based on the reversal of therapeutic resistance are being designed for cancer treatment (Figure 7c). The 4EBP-based therapeutic peptides hinder the progression of ovarian cancer by binding to eIF4E and disrupting tumor growth. At the same time, the anti-cancer effects of the fusion peptide are even more pronounced when this therapeutic peptide is combined with an analog of gonadotropin-releasing hormone (GnRH). The GnRH-4EBP fusion peptide can inhibit ovarian tumor growth in an epithelial ovarian cancer xenograft model without any cytotoxicity [174]. P53 as an oncogene can be inhibited by RBM38 by suppressing eIF53E-mediated translation of P4 mRNA. Another 4EBP-based therapeutic peptide, Pep8, which mimics the eIF8E bonding domain, can antagonize RBM38, thereby promoting P53 expression, reducing cancer development, and slowing down cancer progression [175].

### 5.3. ASO and siRNA-Based Strategies

ASOs (antisense oligonucleotides) and siRNAs are commonly used to regulate gene expression, and both oligonucleotides can bind their target RNAs via Watson-Crick base pairing to regulate splicing, target miRNAs, and inhibit translation. SiRNAs are used to target RBPs to reverse their abnormal expression. SiRNAs can bind to target genes and subsequently cause gene silencing. SiRNA-based therapies have shown efficacy and clinical safety in clinical practice. SiRNA efficacy has been investigated in several tumor types (Figure 7c). SiRNAs targeting eIF4E inhibit tumor growth and stimulate the cytotoxic effects of cisplatin in human breast cancer in vitro and in vivo, suggesting that cisplatin treatment in combination with eIF4E-siRNA therapy would be more successful [176]. HuR can also be targeted with siRNA, a combined chemical-biological drug delivery system that can be used to actively target desired cells or tissues, leading to increased reactive oxidative stress, which increases radiosensitivity and sensitizes TNBC cells to radiation [177]. Furthermore, loading siRNA targeting HuR into folic acid (FA)-coupled polyamide-amine dendrimer (Den)-based nanoparticles revealed that the formulation was effective in reducing HuR expression and cell proliferation in lung cancer cells. In combination with cis-diaminoplatinum (CDDP), this nanoparticle exhibited synergistically improved anticancer effects and reduced cytotoxicity [178]. Similarly, when siRNA was targeted to HuR in a mouse lung cancer model, HuR expression was disrupted and tumor growth was inhibited in a liposomal nanoparticle delivery system [179]. Specific ASOs targeting eIF4E can inhibit tumor growth by suppressing the translation of target mRNAs such as VEGF, Survivin, C-Myc, Cyclin D1, and BCL-2 [137,180,181]. A number of FDA-approved or clinically tested drugs enable gene-specific silencing, and although not yet approved for cancer therapy, many researchers are conducting relevant clinical and non-clinical studies [182]. For example, in the context of targeting RBP, ISIS 183750, an ASO drug targeting eIF4E, disrupts the proliferation of colorectal cancer cells [183]. In addition, ASOs and siRNAs targeting MSI can inhibit tumor growth in pancreatic and ovarian cancers in vitro and in vivo [184,185] (Figure 7d).

### 5.4. Binding Partners and Downstream Effectors

The strategy of targeting RBPs for cancer treatment is not a bad option, but the success rate has been a stumbling block to this strategy due to the difficulty of either direct targeting or specific selection of RBPs. However, it is also reassuring to note that RBPs have great potential to become markers for different types of cancer, both diagnostically and prognostically, due to their aberrant expression profiles in different environments and the variation in their regulatory functions on target mRNAs. Gene regulation of RBP in rodent tumor models has shown exciting inhibitory effects on tumor development in different types of cancer studies, including leukemia. RBP’s therapeutic potential has been extensively studied, as some RBPs have been found to be promising biomarkers of prognosis for patients with different types of cancer [186,187]. For example, the aggressive phenotype or poor prognosis of several cancers has been associated with the upregulation of IGF2BP3 [163,188].

In order to improve the success rate of cancer treatment, many researchers have focused on binding partners of RBPs and downstream effectors of RBPs. With advances in bioinformatics methods and experimental techniques, the veil of RBP-binding chaperones involved in cancer progression is gradually being lifted. Direct targets of various RBPs in different cancer types have been identified through microarray and sequencing techniques [189].

Downstream effectors of RBPs are also good choices for therapeutic targets. Among them, MYC is, on the one hand, a common effector of many RBPs. On the other hand, it can also inversely regulate the stability of several RBPs, including HuR, hnRNPA1, and hnRNPH, to participate in cancer progression. Because of its ability to inversely regulate the function of RBPs, it is possible to intervene in RBP-influenced cancer progression by targeting and regulating the transcriptional levels and activity of MYC. For example, small molecule inhibitors such as I-BET, JQ1, and MMS417 have been identified as transcriptional repressors of MYC, and these small molecule inhibitors can significantly inhibit cancer progression. ASO is another MYC transcriptional repressor that can down-regulate MYC transcriptional levels by reducing MYC translation and splicing events. In addition to suppressing MYC activity, it is also possible to inhibit MYC and its binding partners by mutating MYC and using small molecule inhibitors on the interaction between MYC and its binding partners (Figure 7d).

Here, we summary information on small molecule inhibitors, therapeutic peptides, antisense oligonucleotides (ASOs), siRNAs, binding partners and downstream effectors (Table 2).

## 6. Conclusions and Future Perspectives

### 6.1. Conclusions

Dysregulation of RBPs is observed across multiple cancer types, leading to modifications in the expression of genes associated with tumorigenesis and influencing the progression of cancer. Simultaneously, RBPs possess the capability to either induce or suppress the expression of genes implicated in cancer, thereby contributing to the heterogeneity observed in cancer progression. On the one hand, RBPs govern the delivery of genetic material from DNA to RNA; on the other hand, in the context of cancer, RBPs participate in the energy metabolism of cancer cells and facilitate their evasion of immune surveillance by modulating mRNA stability and the translation process. Therefore, within the field of cancer biology, RBP has great research and therapeutic potential.

Although much has been explored and many researchers have achieved much in identifying RBPs and RBP-RNA interactions, there are still many questions and unknowns that deserve continued in-depth investigation. For example, the use of RBP- RNA-specific binding for targeted drug therapy still requires work. In order to introduce RBP-targeted drugs into cancer therapy, we need better identification of RBPs and a deeper understanding of the target RNAs that interact with them, as well as the signaling pathways and regulatory mechanisms involved in the interactions, so that we can target RBPs accordingly for different cancer patients. Methods such as primer extension (SHAPE), chromatin immunoprecipitation (CHIP), RNA immunoprecipitation (RIP), and Ribosome analysis (Ribo-Seq) can be used to investigate the feasibility and effectiveness of targeted RBP therapy. Therefore, the use of targeted RBP therapy in cancer requires us to explore more deeply in the future to study the interaction between RBPs and RNA.

On the therapeutic side, several researchers have experimentally validated the use of small molecule inhibitors of RBPs, therapeutic peptides, and ASO-specific antagonism of RBPs in vitro, and the experimental results have shown encouraging effectiveness. Nevertheless, questions remain as to whether RBPs can be used as an effective marker for cancer diagnosis, prognosis, and treatment.

### 6.2. Future Perspectives

In summary, we make some suggestions for future RBP research. First, although the mechanical alterations triggered by dysregulated RBP expression have been shown, this is only the tip of the iceberg, and there are still a large number of unidentified mechanistic alterations mediated by RBPs that require more and more in-depth scientific studies. The deeper our understanding of RBP’s dysregulation-mediated mechanical alterations, the better our understanding of RBP’s function will be. Secondly, RBPs act primarily through binding to target RNAs to form RNP complexes and a complex regulatory network that regulates cellular processes through various post-transcriptional mechanisms. A change in one node of the regulatory network can have a butterfly effect, so further deciphering the unknown post-transcriptional mechanisms is urgent. Thirdly, RBP is currently seen mainly in tumor tissues but can be detected by extracting RBPs from blood, urine, and other secretion samples for combined diagnostic testing. Finally, in the process of targeted cancer therapy, how to reduce or avoid the delivery of RBPs-targeted drugs to tumor tissue by the patient’s autoimmune response and how to better ensure the effectiveness and effective amount of drugs are also directions we need to work on. In the future, as our comprehension of RBP-mediated cancer regulatory networks advances, the integration of numerous data resources will encounter substantial challenges. Consequently, the development of comprehensive analysis software pertaining to this domain assumes utmost importance.

## Figures and Tables

**Table 1:** Roles of RNA-binding proteins (RBPs) in cancer.

RBP	The Basic Mechanism of RBPs Regulation	Tumor Type	Biological Functions	References
HuR	Subcellular localization, mRNA stability, mRNA translation	Gastric Cancer, Breast Cancer, Colon Cancer, Lung Cancer, Varian Cancer	Exerts proliferation anti-apoptotic effects	[129,130,131,132]
RBM38	mRNA stability, mRNA translation, post-transcriptional regulation, mRNA splicing	Colorectal Cancer, Acute Myeloid Leukemia, Renal Cell Carcinoma, Hepatocellular Carcinoma	Inhibit EMT, stemness, invasiveness	[133,134,135,136]
eIF4E	Translation	B-Cell Lymphoma, Breast, Colon, Lymphoma, Melanoma	Promotes apoptosis, angiogenesis, EMT, invasion, metastasis	[137,138,139]
hnRNPD (AUF1)	mRNA stability	Breast Cancer, Colon Cancer, Stomach Cancer Liver, Lung, Pancreatic Cancer Sarcoma, Thyroid Cancer	Promotes proliferation, Senescence	[92,140,141,142,143]
hnRNPA2/B1	Alternative splicing	Brain Tumor, Lung Cancer	Promotes proliferation, EMT, metastasis	[122,144,145,146,147,148]
TRBP	mRNA translation, mRNA stability	Breast Carcinomas, Colorectal Cancer, Endometrial Cancer	Promotes or inhibits cell proliferation and invasion	[149,150]
IGF2BP1 (IMP1/ZBP1)	Subcellular localization, mRNA stability	Breast Cancer, Colon Cancer, Lung Cancer, Melanoma, Ovarian Cancer, Skin Cancer, Liver Cancer	Promotes proliferation, EMT, invasion, metastasis	[99,100,110,111,151,152,153,154,155]
IGFBP2 (IMP2)	Subcellular localization, mRNA stability	Breast Cancer, Leukemia, Lung Cancer, Colon Cancer	Promotes EMT, invasion, metastasis	[155,156,157,158]

**Table 2:** RBP-based targeted therapeutic strategies in cancer.

Therapeutic Types	RBP	Therapeutic Approaches	Functions	Tumor Types	References
Small molecule inhibitors	HuR	MS-444, DHTS, AZA-9	Targets RRM1 and RRM2 of HuR and inhibits RNA-binding activities of HuR	Pancreas, Colon, Melanoma, Brain, Breast	[161,162,164,165]
	eIF4E	Ribavirin 4Ei-1	Impedes eIF4E; antagonizes eIF4E cap binding; and initiates degradation	Hscc, Aml, Breast, Lung, Mesothelioma	[168,169]
	MSI-1	(-)-gossypol; Oleic acid	Blocks RBP binding site with RNA; interfere with MSI-1 expression	Brain (CNS) Colon	[171,172]
Therapeutic peptides	eIF4E	GnRH-4EBP	Binds to eIF4E and disrupts eIF4E interacting with eIF4G	Ovary	[175,190]
	RBM38	Pep8	Antagonizes RBM38 and promotes p53 expression	Colon, Breast	
ASO	eIF4E	ISIS 183750	Inhibits the proliferation of cancer cells	Colon	[183]
		VEGF, c-Myc, Survivin, BCL-2 Cyclin D1	Inhibit the translation of target mRNAs to inhibit tumor growth		
siRNA	eIF4E		Stimulates the cytotoxic effects of cisplatin	Breast	[176]
	HuR		Silences HuR expression; interferes with the binding of HuR with mRNA	Lung	[178,179]
Binding partners and downstream effectors	HuR	MYC	Targeted regulation of MYC transcript levels and activity; intervenes in RBP-affected cancer progression		[163,188]
	hnRNPA1				
	hnRNPH				

## Data Availability

Data sharing not applicable.

## Abbreviations

**RBPs**	RNA-binding proteins
**RBDs**	RNA-binding domains
**ncRNAs**	non-coding RNAs
**lncRNA**	long non-coding RNA
**miRNAs**	microRNAs
**hnRNPK**	heterogeneous nuclear ribonucleoprotein K
**lncRNAs**	long-stranded non-coding RNAs
**dsRBD**	double-stranded RNA binding structures
**dsRNA**	double-stranded RNA
**siRNAs**	small interfering RNAs
**snRNPs**	small nuclear proteins
**snoRNA**	small nucleolar RNA
**snRNA**	small nucleolar RNA
**TTP**	Tritetraproline
**AUF1**	AU-rich elemental RNA-binding protein 1
**HuR**	Human antigen R
**IGF2BP**	Insulin-like growth factor 2 mRNA-binding protein
**UTRs**	Untranslated regions
**CRC**	Colorectal cancer
**ESRP1**	Epithelial splicing regulatory protein 1
**RBM38**	RNA-binding motif protein 38
**m7G**	7-methylguanosine
**AUBP**	ARE-binding protein
**MSI1**	Musashi RNA-binding protein 1
**eIF4E**	eukaryotic translation initiation factor 4E
**RNPs**	Ribonucleoprotein complexes
**RRM**	RNA Recognition Motifs
**KH**	K homology structures domain
**ZnFs**	Zinc finger structural domains
**DsRBDs**	Double-stranded RNA-binding structural domains
**AS**	Alternative splicing
**APA**	Alternative polyadenylation
**CPA**	Cleavage and polyadenylation
**CPE**	Cytoplasmic polyadenylation elements
**APC**	Adenomatous polyposis protein
**EMT**	Epithelial-mesenchymal transition
**3′UTR**	3′untranslated regions
**5′UTR**	5′untranslated regions
**ASOs**	Antisense oligonucleotides
